# Determinants of non-utilization of health facilities for childbirth in Papua New Guinea: Evidence from the demographic and health survey

**DOI:** 10.1371/journal.pone.0338606

**Published:** 2025-12-30

**Authors:** McKenzie Maviso, Gracelyn Potjepat, Rebecca Bogarobu Emori, Paula Zebedee Aines, Carolyn Ruth Hastie

**Affiliations:** 1 Division of Public Health, School of Medicine and Health Sciences, University of Papua New Guinea, Port Moresby, National Capital District, Papua New Guinea; 2 School of Social Sciences, Faculty of Arts, Social Sciences and Humanities, University of Wollongong, Northfields Ave, Wollongong, New South Wales Australia; 3 Division of Nursing, School of Medicine and Health Sciences, University of Papua New Guinea, Port Moresby, Papua New Guinea; 4 Early Start Research Institute, University of Wollongong, Northfields Ave, Wollongong, New South Wales Australia; 5 Population Health Unit, Papua New Guinea Institute of Medical Research, Goroka, Eastern Highlands Province, Papua New Guinea; 6 School of Nursing and Midwifery, Griffith University, Logan Campus, University Drive, Meadowbrook, Qld, Australia; Menzies School of Health Research: Charles Darwin University, AUSTRALIA

## Abstract

**Background:**

Health facility-based childbirth services are essential for reducing maternal and neonatal mortality. Yet, these services remain underutilized in many low- and middle-income countries (LMICs), including Papua New Guinea (PNG), where birthing at home or in village settings continue to predominate. This study investigated the determinants of non-utilization of health facilities for childbirth among women in PNG.

**Methods:**

Data from a weighted sample of 6,432 women using the 2016–2018 PNG Demographic and Health Survey (DHS) were analyzed. Descriptive statistics and multivariate logistic regression analyses were conducted to determine the factors associated with the non-utilization of health facilities for childbirth. The adjusted odds ratios (AOR) with their corresponding 95% confidence intervals (CI) were computed using the Statistical Package for Social Sciences, version 30.0.

**Results:**

Overall, 58.3% (95% CI: 57.0–59.5) of women did not give birth in a health facility. Women were more likely to give birth at home or in the village if they had no formal education (AOR: 1.48; 95% CI: 1.11–2.09), lived in rural areas (AOR: 1.31; 95% CI: 1.11–1.75), were from the Southern (AOR: 1.05; 95% CI: 1.01–1.54), or Momase (AOR: 1.06; 95% CI: 1.02–1.83) regions, made their own healthcare decisions (AOR: 1.21; 95% CI: 1.10–4.14), had these decisions made by their husbands (AOR: 1.69; 95% CI: 1.13–2.63), walked to a health facility (AOR: 1.01; 95% CI: 1.00–1.67), or traveled more than 24 hours to access care (AOR: 1.02; 95% CI: 1.39–2.70), and had no antenatal care visits during pregnancy (AOR: 1.08; 95% CI: 1.04–1.51).

**Conclusions:**

Over half of the women in this study did not utilize health facilities for childbirth. Demographic and health service-related factors influenced the non-utilization of facility-based childbirth, highlighting the need to scale up maternal health services. Increasing uptake of facility-based childbirth requires coordinated system-level efforts and incentive-based interventions that promote antenatal care and skilled birth attendance, especially for rural women. Male-inclusive strategies in maternal health decision-making are critical for improving women’s access to and utilization of facility-based childbirth services.

## Introduction

Maternal mortality remains a significant global health challenge, disproportionately concentrated in many low- and middle-income countries (LMICs), where systemic health inequities are more deeply entrenched [1]. Globally, approximately 800 women die each day from complications related to pregnancy and childbirth, reflecting a maternal mortality ratio (MMR) of 223 deaths per 100,000 live births [2]. Despite substantial global initiatives to strengthen maternal healthcare, progress remains limited and uneven, with preventable maternal deaths still considerably high, particularly among socioeconomically marginalized women [3]. In 2023, approximately 260,000 women died from preventable causes associated with pregnancy and childbirth, almost 92% of whom died in LMICs [4]. The leading cause of maternal death globally is hemorrhage (27%), followed by indirect obstetric causes (23%), hypertensive disorders (16%), and puerperal sepsis (15%) [4–6]. Enhancing access to high-quality, affordable maternal health services is essential for reducing preventable maternal deaths.

The presence of skilled birth attendants is consistently recognized as a critical determinant in the reduction of maternal and neonatal mortality [7,8]. According to the World Health Organization (WHO), a skilled birth attendant is a certified health professional, such as a midwife, doctor, or nurse, with the necessary education and training to provide effective care and support to women during normal childbirth and the immediate postnatal period, as well as to identify and attend to obstetric complications [9,10]. Supervised births in health facilities by skilled birth attendants align with the United Nations Sustainable Development Goal 3 to reduce maternal and neonatal mortality and improve the survival rates of mothers and newborns by 2030 [11,12]. Recent global estimates from Joint UNICEF-WHO data indicate that the number of skilled birth attendants has improved from 61% in 2000 to 86% in 2023 [13]. Evidence indicates that timely access to skilled care and emergency obstetric services could prevent up to 75% of maternal deaths globally [4].

Despite global improvements in skilled birth attendant coverage, many women continue to give birth without their support, even where such services are accessible [7,13]. A comparative study found that a significant proportion of women in Nigeria (54.9%) and Ethiopia (45.4%) preferred home or village births due to the perception that facility-based childbirth was unnecessary [14]. A population-based survey found that nearly half (47.6%) of Guinean women did not give birth at health facilities [15]. Women’s choice of birthplace is shaped by a complex set of factors, including maternal characteristics (e.g., age, parity, education, and marital status), household attributes (e.g., family size, wealth, and socioeconomic status), geographic barriers (e.g., rural residence and long distances to health facilities), sociocultural norms and beliefs (e.g., traditional birthing practices and gender roles), and health service-related factors (e.g., poorly maintained infrastructure, negative staff attitudes, and limited trust in formal care) [15–18].

Papua New Guinea (PNG) has one of the world’s most geographically dispersed and isolated populations, creating significant health challenges driven by limited accessibility, systemic inequities, and a fragmented healthcare system [19,20]. The country also has one of the highest maternal mortality rates in the Western Pacific region, estimated at 189 per 100,000 live births [21]. In the absence of reliable, consistent country-specific data, international organizations, such as the United Nations Maternal Mortality Estimation Inter-Agency Group (MMEIG) produce estimates to address these gaps [21]. However, the accuracy of these estimates remains contested, as evidenced by national data sources (e.g., Demographic and Health Surveys and Health Information Systems) and local research, which often report significantly higher maternal mortality figures than those produced by internationally applied statistical models [22]. Despite the challenges in measurement, the MMR in PNG remains unacceptably high. Most maternal deaths in the country are attributed to obstetric hemorrhage, sepsis, embolism, eclampsia, and unsafe abortion, conditions that are mostly preventable through appropriate and timely maternal healthcare [23–25]. Sociodemographic factors, along with the absence of skilled or supervised care during antenatal visits, childbirth, and the early postpartum period, are key contributors to these preventable deaths [24,25]. Ensuring equitable, high-quality access to antenatal care and skilled birth attendance is essential for improving maternal and neonatal health outcomes.

Over the past decade, the PNG government and its development partners have made substantial investments to improve maternal health through collaborative initiatives. However, implementation has remained disproportionate and hindered by persistent challenges [19,26]. In 2023, only about 45% of births occurred in health facilities in PNG [27], indicating critically low access to and utilization of maternal healthcare services, including supervised births by skilled birth attendants [18,28]. A recent study revealed that just over half of women in PNG accessed antenatal care (52.3%) and received support from skilled birth attendants (58.7%), while postnatal care use remained low, with only 26.6% of women accessing these services [28]. While women who access antenatal care and give birth in health facilities often acknowledge the benefits of these services, their decisions to seek maternal healthcare are influenced by a complex interplay of factors [29]. A maternal and infant health survey across three provinces in PNG found that 44% of home or village births occurred among multiparous women with limited formal education [30]. Contributing factors to the avoidance of facility-based childbirth include sociocultural beliefs, inadequate health infrastructure, negative staff attitudes, and apprehension with male birth attendants [30–32]. Significant disparities in maternal health pose a major challenge to public health policy and service delivery, underscoring the need for urgent, targeted interventions. This study investigated the prevalence and determinants of non-utilization of health facilities for childbirth among women in PNG, drawing on nationally representative survey data.

## Materials and methods

### Data source and sampling

Data were drawn from the 2016–2018 PNGDHS, a population-based cross-sectional survey. The DHS collects national data on maternal and child health, such as fertility, contraceptive use, parity, breastfeeding and infant feeding practices, children’s nutritional status, immunization, women’s empowerment, intimate partner violence, awareness and behavior regarding HIV/AIDS and other sexually transmitted infections (STIs), and other health-related factors [33]. The survey used the list of census units (CU) from the 2011 National Population and Housing Census as the sampling frame [34].

The PNGDHS uses a two-stage stratified sampling technique. In the first stage, 800 CU were selected, which was achieved through a probability proportional to the CU size [33]. The second sampling stage involved selecting 24 households from each cluster via equal probability systematic selection, resulting in a sample of approximately 19,200 households. In the survey, 18,175 women of reproductive age were identified, and 15,198 completed their interviews, resulting in an 84% response rate. This study included a weighted sample of 6,432 women aged 15–49 years who had complete information on the variables of interest and had given birth in the three years preceding the survey. Details of the methodology, pretesting, training of field workers, sampling design, and selection are available in the final DHS report [33]. The dataset is publicly accessible online via this link: https://dhsprogram.com/methodology/survey/survey-display-499.cfm.

### Study variables and measurements

The dependent variable for this study was the place of birth. During data collection, respondents were asked, “Where did you give birth to (NAME)?” The responses included home or village (i.e., respondents’ home or in the village), public sector (i.e., government hospital, health center, or community health post), and private sector (i.e., church hospital or health center, community health post, or private hospital/clinic or other private health facilities). A new variable for place of birth was created and coded as ‘0’ to indicate childbirth at home or in a village, and ‘1’ to indicate childbirth in a health facility.

The explanatory variables included in this study were selected based on their availability in the dataset and previous studies associated with the use of skilled birth attendants or supervised births in health facilities [15,18,35,36]. These variables comprised age, marital status, respondent’s educational level, husband’s educational level, respondent’s employment status, husband’s employment status, wealth index, administrative region, place of residence, sex of the household head, decision-making for healthcare services, health insurance coverage, mode of transport to reach a health facility, distance to reach a health facility, number of antenatal care visits, and the number of children alive (**Table 1**).

**Table 1:** Demographic characteristics of the participants, PNGDHS 2016–2018.

Variables	*N* = 6,432	%
**Age (years)**		
15–24	1,625	25.3
25–34	3,063	47.6
35–44	1,568	24.4
45 or more	176	2.7
**Marital status**		
Married	6,227	96.8
Cohabiting	206	3.2
**Respondent’s educational level**		
No formal education	1,567	24.4
Primary	3,203	49.8
Secondary	1,389	21.6
Higher	273	4.3
**Husband’s education level (missing, *n* = 790)**		
No formal education	1,119	19.8
Primary	2,422	42.9
Secondary	1,627	28.8
Higher	474	8.4
**Respondent’s employment status**		
Not working	4,420	68.7
Working	2,012	31.3
**Husband’s employment status (missing, *n* = 931)**		
Not working	2,738	49.8
Working	2,763	50.2
**Wealth index**		
Poorer	1,301	20.2
Poor	1,268	19.7
Middle	1,282	19.9
Rich	1,321	20.5
Richer	1,260	19.6
**Region**		
Southern	1,273	19.8
Highlands	2,457	38.2
Momase	1,773	27.6
Islands	929	14.4
**Place of residence**		
Urban	748	11.6
Rural	5,684	88.4
**Sex of the household head**		
Male	5,426	84.3
Female	1,007	15.7
**Decision-making for healthcare service (missing, *n* = 695)**		
Respondent alone	1,671	29.1
Joint decision	3,193	55.6
Husband/partner	769	13.4
Someone else	104	1.8
**Covered by health insurance**		
No	6,245	97.1
Yes	187	2.9
**Mode of transport to the health facility (missing, *n* = 4,078)**		
On foot	1,293	54.9
Vehicle	926	39.4
Boat/canoe	134	5.7
**Travel distance to the nearest health facility (hours) (missing, *n* = 4,068)**		
0–5	1,333	56.4
6–23	958	40.5
24 or more	73	3.1
**Number of antenatal care visits (missing, *n* = 313)**		
None	1,367	22.3
1–3	1,517	24.8
4 or more	3,235	52.9
**Number of children living**		
1	1,565	24.3
2	1,325	20.6
3 or more	3,542	55.1

**Note:** Weighted frequencies (*N*) and percentage (%).

### Statistical analysis

All statistical analyses were computed using the Statistical Package for the Social Sciences (SPSS) for Windows, version 30.0 (Armonk, NY: IBM Corp., 2023). Descriptive statistics were conducted to obtain valid weighted frequencies and percentages. Pearson’s chi-square test of independence was used to examine the association between the outcome variable and the explanatory variables. Those variables that were statistically significant at an alpha level of 0.05 were included in the multivariate logistic regression analysis. A test for multicollinearity was conducted via the variance inflation factor (VIF) to assess the correlations among the independent variables [37]. The results revealed no multicollinearity (the VIF ranged from 1.03 to 1.32, with a mean of 1.31). The relationship between the explanatory variables and the place of birth was assessed using adjusted odds ratios (AOR) with their 95% confidence intervals (CI). Sample weight (women’s sample weight by 1,000,000) was applied to account for the unequal sampling of women from the enumeration areas, and the complex samples plan (‘cplan’) for logistic regression in SPSS was used in the analysis to account for the survey’s complex nature. The primary sampling units, sample strata, and sample weights were adjusted in the univariate, bivariate, and multivariate analyses. This enhanced the accuracy of the CI and standard errors in the multivariate logistic regression model. Statistical significance was set at *p* < 0.05. This study adhered to the guidelines outlined in the Strengthening the Reporting of Observational Studies in Epidemiology (STROBE) statement [38].

### Ethics consideration

Ethics approval was not required for this study, as it used publicly available secondary data obtained from the DHS Program website (https://dhsprogram.com/). Informed consent was obtained from each participant before the survey, and the anonymity and confidentiality of the participants were ensured. All the methods were conducted in accordance with the relevant guidelines and regulations.

## Results

### Characteristics of the study participants

**Table 1** presents the sociodemographic characteristics of the study participants. Among the total 6,432 women (weighted sample), the mean age was 29.89 years (SD = 7.01), with nearly half aged between 25 and 34 years (47.6%) and had a primary education level (49.8%). More than two-thirds of the participants were not working (68.7%) and lived in rural areas (88.4%). Similarly, over half of the respondents and their husbands made joint decisions regarding healthcare services (55.6%), had to travel for approximately five hours to reach the health facility (56.4%), and had four or more antenatal care visits (52.9%).

### Bivariate analysis of place of birth for women in PNG

**Table 2** presents the bivariate analysis of factors associated with the place of birth in PNG. Overall, 3,809 women (58.3% [95% CI: 57.0–59.5]) reported giving birth either at home or in the village. Among these respondents, more than half were aged 15–24 years (66.3%), married (59%), had a primary education level (57.2%), were unemployed (56%), and lived in rural areas (55.4%). Nearly two-thirds (63%) made their own decisions regarding healthcare services. Three-quarters reported walking for about five hours to reach a health facility (76.6%) and having four or more antenatal care visits (76.5%). Chi-square analysis results indicated a statistically significant association between all explanatory variables, except marital status, and the non-utilization of health facilities for childbirth among women in PNG (*p* < 0.05).

**Table 2:** Bivariate analysis of place of birth for women in PNG, PNGDHS 2016–2018.

Variables	Facility-based birth	%	Home or village birth	%	*p*-value*
**Sample (*N*)**	**2,623**	**41.7**	**3,809**	**58.3**	
**Age (years)**					< 0.001
15–24	548	33.7	1,077	66.3	
25–34	1,268	41.4	1,795	58.6	
35–44	703	44.8	865	55.2	
45 or more	105	59.3	72	40.7	
**Marital status**					0.061
Married	2,552	41.0	674	59.0	
Cohabiting	71	34.5	135	65.6	
**Respondent’s educational level**					< 0.001
No formal education	1,017	64.9	550	35.1	
Primary	1,371	42.8	1,832	57.2	
Secondary	228	16.4	1,161	83.6	
Higher	7	2.6	267	97.4	
**Husband’s educational level (missing, *n* = 790)**					< 0.001
No formal education	717	64.1	402	35.9	
Primary	1,196	49.4	1,225	50.6	
Secondary	376	32.1	1,251	76.9	
Higher	60	12.7	414	87.3	
**Respondent’s employment status**					< 0.001
Not working	1,943	44.0	2,477	56.0	
Working	680	33.8	1,332	66.2	
**Husband’s employment status (missing, *n* = 931)**					< 0.001
Not working	1,343	49.1	1,395	50.9	
Working	942	34.1	1,821	65.9	
**Wealth index**					< 0.001
Poorer	850	65.3	451	34.7	
Poor	751	59.2	517	40.8	
Middle	556	43.4	726	56.6	
Rich	374	28.3	947	71.7	
Richer	91	7.2	1,169	92.8	
**Region**					< 0.001
Southern	399	31.4	873	68.6	
Highlands	1,013	41.2	1,444	58.8	
Momase	980	55.2	794	44.8	
Islands	232	24.9	698	75.1	
**Place of residence**					< 0.001
Urban	90	12.0	658	88.0	
Rural	2,533	44.6	3,151	55.4	
**Sex of the household head**					< 0.001
Male	2,320	42.8	3,106	57.2	
Female	303	30.1	704	69.9	
**Decision-making for healthcare service (missing, *n *= 695)**					< 0.001
Respondent alone	619	37.0	1,052	63.0	
Joint decision	1,309	41.0	1,884	59.0	
Husband/partner	389	50.6	380	49.4	
Someone else	57	54.8	47	45.2	
**Covered by health insurance**					< 0.001
No	2,609	41.8	3,636	58.2	
Yes	14	7.5	173	92.5	
**Mode of transport to the health facility (missing, *n* = 4,078)**					< 0.001
On foot	519	40.1	774	59.9	
Vehicle	221	23.8	706	76.2	
Boat/canoe	46	34.3	88	65.7	
**Travel distance to the nearest health facility (hours) (missing, *n* = 4,068)**					< 0.001
0–5	312	23.4	1,021	76.6	
6–23	429	44.7	530	55.3	
24 or more	43	58.9	30	41.1	
**Number of antenatal care visits (missing, *n* = 313)**					< 0.001
None	1,183	86.5	184	13.5	
1–3	576	38.0	941	62.0	
4 or more	759	23.5	2,477	76.5	
**Number of children living**					< 0.001
1	430	27.5	1,135	72.5	
2	466	35.2	859	64.8	
3 or more	1,727	48.8	1,815	51.2	

*Chi-square test; *p* ≤ 0.05.

### Determinants of the non-utilization of health facilities for childbirth

**Table 3** presents the determinants of the non-utilization of health facilities for childbirth among women in PNG. Multivariate analysis showed that women were more likely to give birth at home or in the village if they had no formal education (AOR: 1.48; 95% CI: 1.11–2.09), lived in rural areas (AOR: 1.31; 95% CI: 1.11–1.75), were from the Southern (AOR: 1.05; 95% CI: 1.01–1.54), or Momase (AOR: 1.06; 95% CI: 1.02–1.83) regions, made their own healthcare decisions (AOR: 1.21; 95% CI: 1.10–4.14), had these decisions made by their husbands (AOR: 1.69; 95% CI: 1.13–2.63), walked to a health facility (AOR: 1.01; 95% CI: 1.00–1.67), or traveled more than 24 hours to access care (AOR: 1.02; 95% CI: 1.39–2.70), and had no antenatal care visits during pregnancy (AOR: 1.08; 95% CI: 1.04–1.51). Conversely, women were less likely to give birth at home or in the village if they were from the Highlands region (AOR: 0.34; 95% CI: 0.19–0.59), had primary (AOR: 0.69; 95% CI: 0.30–0.87), or secondary education (AOR: 0.11; 95% CI: 0.03–0.43), made joint healthcare decisions (AOR: 0.83; 95% CI: 0.24–0.96), traveled by vehicle (AOR: 0.25; 95% CI: 0.10–0.58), reached a health facility within 24 hours (AOR: 0.72; 95% CI: 0.49–0.93), and attended at least three antenatal care visits (AOR: 0.49; 95% CI: 0.34–0.69).

**Table 3:** Determinants of non-utilization of health facilities for childbirth among women in PNG, PNGDHS 2016–2018.

Variables	Crude OR	95% CI	Adjusted OR	95% CI	*p*-value*
**Age (years)**					0.256
15–24	Ref.		Ref.		
25–34	0.72	0.59–0.88	1.56	0.60–4.07	
35–44	0.63	0.51–0.77	1.14	0.47–2.77	
45 or more	0.35	0.22–0.56	1.54	0.64–3.73	
**Respondent’s educational level**					< 0.001
No formal education	0.81	0.5–1.14	1.48	1.11–2.09	
Primary	0.15	0.09–0.25	0.69	0.17–0.95	
Secondary	0.48	0.29–0.78	0.11	0.03–0.43	
Higher	Ref.		Ref.		
**Husband’s educational level**					0.051
No formal education	0.08	0.05–0.14	0.46	0.19–0.63	
Primary	0.15	0.09–0.25	0.69	0.30–0.87	
Secondary	0.48	0.29–0.78	0.95	0.43–1.00	
Higher	Ref.		Ref.		
**Respondent currently working**					0.379
Not working	0.65	0.51–0.83	0.84	0.57–0.94	
Working	Ref.		Ref.		
**Husband’s employment status**					0.851
Not working	0.54	0.44–0.65	1.03	0.73–1.47	
Working	Ref.		Ref.		
**Wealth index**					0.080
Poorer	0.04	0.03–0.06	0.26	0.12–0.59	
Poor	0.05	0.04–0.08	0.22	0.09–0.53	
Middle	0.10	0.07–0.15	0.33	0.15–0.76	
Rich	0.19	0.13–0.29	0.38	0.17–0.87	
Richer	Ref.		Ref.		
**Region**					< 0.001
Southern	0.73	0.50–1.05	1.05	1.01–1.54	
Highlands	0.47	0.33–0.68	0.34	0.19–0.59	
Momase	0.27	0.17–0.42	1.06	1.02–1.83	
Islands	Ref.		Ref.		
**Place of residence**					0.010
Urban	Ref.		Ref.		
Rural	0.71	0.50–1.05	1.31	1.11–1.75	
**Sex of the household head**					0.499
Male	0.58	0.45–0.74	0.82	0.46–0.97	
Female	Ref.		Ref.		
**Decision-making for healthcare service**					0.024
Respondent alone	2.04	0.61–6.83	1.21	1.10–4.14	
Joint decision	1.72	0.52–5.69	0.83	0.24–0.96	
Husband/partner	1.17	0.34–4.01	1.69	1.13–2.63	
Someone else	Ref.		Ref.		
**Covered by health insurance**					0.320
No	0.12	0.04–0.33	0.54	0.16–0.82	
Yes	Ref.		Ref.		
**Mode of transport to the health facility**					0.038
On foot	0.78	0.48–1.27	1.01	1.00–1.67	
Vehicle	0.68	0.39–1.85	0.25	0.10–0.58	
Boat/canoe	Ref.		Ref.		
**Travel distance to the nearest health facility (hours)**					0.021
0–5	Ref.		Ref.		
6–23	2.65	1.87–3.74	0.72	0.49–0.93	
24 or more	4.74	2.38–9.46	1.02	1.39–2.70	
**Number of antenatal care visits**					< 0.001
None	0.61	0.41–0.80	1.08	1.04–1.51	
1-3	0.50	0.39–0.64	0.49	0.34–0.69	
4 or more	Ref.		Ref.		
**Number of children living**					0.326
1	Ref.		Ref.		
2	0.69	0.56–0.87	1.40	0.84–2.35	
3 or more	0.39	0.32–0.49	1.41	0.84–2.35	

*Statistical significance; *p* ≤ 0.05.

Ref. = reference category; OR = odds ratio; CI = confidence intervals.

## Discussion

Drawing on a nationally representative sample, this study examined the determinants of non-utilization of health facilities for childbirth in PNG. Findings revealed that more than half (58.3%) of the women did not give birth in a health facility. The prevalence of home or village births in PNG is relatively high compared with previous findings in Guinea (47.6%) [15] and Tanzania (21.4%) [7]. However, substantially higher rates of home or village births have been reported in several countries, including Chad (78%), Ethiopia (73%), and both Niger and Yemen (70%) [39]. Reported variations in the prevalence of home or village births across these studies may be attributed to differences in study populations, geographic contexts, and methodological approaches. The persistently high rate of non-facility births in PNG reflects systemic inequities in access to maternal healthcare services, especially skilled care during childbirth [18]. Consequently, this presents a significant challenge to maternal and child health, as it is closely associated with higher rates of maternal and infant mortality.

The findings indicate an inverse correlation between women’s educational level and the non-utilization of health facilities for childbirth. Women with lower educational levels are significantly less likely to give birth in a health facility than their more educated counterparts, indicating that education may be a key determinant of health-seeking behavior during pregnancy and childbirth. This finding corroborates recent studies from India and PNG [18,40,41]. Women with higher levels of education tend to possess better knowledge of available services and have greater autonomy in seeking healthcare services [28,40]. Education plays a critical role in enhancing women’s understanding of maternal health services and in fostering informed decision-making about their own health. Strategic investment in reproductive health education, both within school curricula and through adult literacy initiatives, can significantly improve the utilization of maternal health services [42,43].

A significant relationship was observed between the place of residence and non-utilization of health facilities for childbirth. Consistent with prior studies [18,31,41,44], the likelihood of rural women giving birth at home or in village settings remains higher than their urban counterparts. Many rural women face significant challenges related to cultural expectations, costs of transport, baby supplies, and accommodation when accessing health facilities. Incentives such as baby bundles (e.g., cotton nappies, blanket, sanitary supplies, and birthing fee), transport vouchers, or food allowance help reduce these burdens, making facility-based childbirth more feasible and appealing [45–47]. Incentive-based interventions may help shift entrenched cultural norms that favor home or village births by reinforcing the social legitimacy of facility-based births. When women receive visible benefits, such as clean birth kits, baby bundles, or public recognition, these practices can become normalized within the community, gradually increasing their acceptability and uptake [45,47].

In this study, women from the Momase and Southern regions had lower odds of facility-based childbirth than those in the Islands region. In these regions, women rely on rural health facilities as their main source of care; yet these facilities frequently operate with limited resources and face chronic challenges stemming from inadequate infrastructure and ongoing logistical barriers [28]. These constraints impede the delivery of essential health services, restrict patient mobility, and contribute to delays in accessing skilled care, particularly during childbirth [18,28]. Another possible reason could be the lack of trained health workers or malfunctioning health facilities, which prevent women from using childbirth services [18,32]. This finding may also reflect the complex interplay of sociocultural norms around childbirth, financial barriers, and prior maternity care experiences that shape mothers’ decisions to give birth at home or within their village [31,32]. Rural–urban gaps in maternal healthcare demand urgent investment in rural health systems to address geographic and infrastructure barriers limiting access for rural women.

Distance from health services was significantly associated with non-utilization of health facilities for childbirth in this study. Women residing farther from health facilities were less likely to use them for childbirth, consistent with previous findings from PNG [28,44]. Geographic challenges, including inadequate road infrastructure and prolonged travel times to health facilities, present substantial barriers for women to access maternal healthcare in the country [31,48,49]. These challenges, often involving navigation through difficult terrain, rivers, forests, and unsafe roads, may help explain why some women give birth at home or in their villages. Moreover, the high cost of transportation and the lack of local healthcare infrastructure also complicate access to health services, resulting in lower motivation among women to use them [28,48]. Improving access to health facilities requires better infrastructure, including enhanced connections to major roads, improved road surfaces, increased transport availability, and affordable transportation options in geographically underserved settings.

Consistent with previous studies [50,51], women without antenatal care visits were more likely to give birth at home or in the village than those who had antenatal visits. Poor uptake of antenatal care services among women may stem from several factors, such as sociodemographic status, the unavailability and inaccessibility of services, a shortage of health workers, and geographic remoteness [49,52]. Women may lack adequate information and education regarding the optimal benefits of antenatal care, which usually encompasses birth preparedness and readiness for complications during pregnancy [52,53]. Social norms and beliefs about pregnancy, labor, birth, and infant feeding practices can influence the uptake of maternal healthcare services [31,54], which could be another explanation for women’s decision to forego antenatal care services to give birth in their homes or villages. As recommended by the WHO, at least eight antenatal care visits for pregnant women remain crucial for improving maternal and neonatal health outcomes and ensuring that women are informed about their health and prepared for childbirth [55]. Enhancing antenatal care uptake requires a comprehensive, multidimensional strategy that addresses geographic, socioeconomic, educational, and cultural determinants.

An inverse association was observed between autonomous decision-making and health facility utilization. Women who made their own healthcare decisions were more likely to give birth at home or in village settings. Such decisions may be influenced by factors such as poor health facility services, previous adverse or traumatic experiences, and a lack of supportive care from health workers [56–58]. These experiences reveal systemic gaps in respectful maternity care and a prevailing mistrust of formal health services. Another explanation could be that some women may opt for home or village births due to the presence of strong social and familial support, often lacking in health facilities. Women with strong familial support and greater autonomy in decision-making regarding childbirth were more likely to avoid health facilities [59].

Additionally, the study’s findings highlight that husbands play a crucial role in shaping women’s decisions regarding the non-utilization of health facilities for childbirth. Several studies have corroborated this finding [44,60,61]. Male involvement in decision-making regarding maternal health services is a critical yet often overlooked factor influencing maternal and neonatal outcomes in PNG [61]. Limited male involvement during pregnancy and childbirth is often driven by sociocultural norms, role conflicts, and a lack of knowledge, resulting in poor decision-making and reduced emotional, financial, and logistical support for women [62,63]. This lack of involvement can delay care-seeking and increase the risks of adverse maternal and neonatal outcomes. However, when husbands and partners actively participate in maternal health services, women are more likely to access timely and appropriate care [62,64]. Strengthening their involvement requires tailored community-based efforts that promote both the benefits of participation and address barriers to informed decision-making. The study emphasizes the urgent need to increase the awareness of facility-based childbirth and enhance institutional support services to improve maternal health outcomes in PNG.

### Strengths and limitations of the study

This study demonstrates several methodological strengths. This population-based survey used a large, nationally representative sample and rigorously validated, standardized data collection protocols to ensure the reliability and generalizability of the findings. The substantial sample size contributed to a high response rate (84%), enhancing the reliability of the findings. Complex sample analysis was also conducted using sampling weights derived from the survey’s multistage stratified design, ensuring accurate estimation of standard errors and confidence intervals. However, the study has some notable limitations. First, the study design was cross-sectional; hence, it is not possible to draw causal inferences from the findings. Second, the survey relied on women’s self-reported health information, which significantly heightened the risk of recall bias. Third, the DHS data do not capture the sociocultural norms and customary practices that influence birth location decisions in rural PNG. Home or village birth often reflects entrenched community and cultural expectations [31,44], which quantitative analyses may overlook. Therefore, the findings should be interpreted with caution, acknowledging this contextual gap. Finally, the absence of qualitative data limits the understanding of critical social, cultural, and systemic factors, such as norms, beliefs, gender-based violence, and health system barriers that may influence women’s decisions around facility-based childbirth. Future qualitative research is essential to elucidate these factors and guide the development of more contextually responsive policies and practices to increase women's use of health facilities for childbirth.

## Conclusions

More than half of the women in this study gave birth at home or in the village settings. The findings identified key demographic and service-related factors, such as maternal education, place of residence, region, decision-making autonomy, distance to health facilities, and frequency of antenatal care visits, as significant determinants of home or village births. Increasing uptake of facility-based childbirth requires coordinated health system efforts and incentive-based interventions to promote antenatal care and skilled birth services, particularly among rural women facing significant socioeconomic and geographic barriers. Furthermore, gender-inclusive strategies that actively engage husbands and partners in reproductive and maternal health decision-making are critical for improving women’s access to and utilization of health facilities for supervised childbirth.

## Supporting information

S1 File STROBE checklist v2. (DOCX)
